# Complexity of Damage-Associated Molecular Pattern Molecule Expression Profile in Porcine Brain Affected by Ischemic Stroke

**DOI:** 10.3390/ijms26083702

**Published:** 2025-04-14

**Authors:** Dominika Golubczyk, Aleksandra Mowinska, Piotr Holak, Piotr Walczak, Miroslaw Janowski, Izabela Malysz-Cymborska

**Affiliations:** 1 Ti-com LLC, Władysława Trylińskiego 2, 10-683 Olsztyn, Poland; dominikagk11@gmail.com; 2 Department of Neurology and Neurosurgery, Collegium Medicum, School of Medicine, University of Warmia and Mazury in Olsztyn, Warszawska 30, 10-082 Olsztyn, Poland; a.zlotkowska@o2.pl; 3 Department of Surgery and Radiology with Clinic, Faculty of Veterinary Medicine, University of Warmia and Mazury in Olsztyn, Oczapowskiego 14, 10-719 Olsztyn, Poland; piotr.holak@uwm.edu.pl; 4 Program in Image Guided Neurointerventions, Department of Diagnostic Radiology and Nuclear Medicine, University of Maryland, 670 W. Baltimore Street, Baltimore, MD 21201, USA; piotrzbaltimore@gmail.com (P.W.); miroslaw.janowski@som.umaryland.edu (M.J.)

**Keywords:** DAMPs, stroke, pig, animal model, brain

## Abstract

Studies using large animal models are essential for better understanding the molecular processes underlying neurological diseases, including ischemic stroke, and serve as a robust foundation for evaluating potential therapies. To better understand the complex role of damage-associated molecular pattern molecules (DAMPs) after ischemia, we aimed to determine their expression in the porcine brain affected by ischemic stroke at four time points: 6 h, 24 h, 3 days and 7 days post-stroke. Within the first 24 h after the stroke, we observed the increased expression of several key factors, including calcium-binding proteins, peroxiredoxins, heat shock proteins and interleukins (*1α* and *1β*, *IL10*, *IL17α*). Moreover, by day 7, multiple DAMPs were up-regulated, coinciding with an enhanced expression of vascular endothelial growth factor A (*VEGFA*) in the affected hemisphere. The effects of ischemic stroke were also evident systemically, as indicated by the altered serum levels of both pro- and anti-inflammatory interleukins, reflecting dynamic inflammatory response. To conclude, our findings provide new insights about the time-dependent DAMP activity in a large animal model of ischemic stroke, highlighting the simultaneous occurrence of an ongoing inflammatory response and the possible initiation of vascular remodeling as early as one week after stroke onset.

## 1. Introduction

Ischemic stroke is one of the three most common causes of death worldwide. An interruption of blood supply through the cerebral arteries causes ischemic changes in the area supported by the occluded vessel and as a consequence of ischemia and a lack of nutrient supply, cells in the brain start to degenerate on the way to necroptosis [1]. Necroptotic neurons produce enhanced increased amount of damage-associated molecu-lar pattern molecules (DAMPs), which increased systemic levels were related to worse functional outcome in human post ischemic stroke [2]. The secretion of individual DAMPs is often interconnected, functioning through feedback mechanisms that amplify the inflammatory response. High-mobility group protein (B)1 (HMGB1) has been identified as an early marker of a bad prognosis after stroke [3]. Shortly after ischemia onset, cells within the lesion release ATP, exacerbating brain damage by triggering cell death pathways [4]. Similarly, as soon as 12 h after ischemic stroke onset, the accumulation of reactive oxygen species leads to the release of peroxiredoxins (PRDXs) from necrotic cells. PRDXs cause the induction of pro-inflammatory cytokine secretion and subsequently the support of immune cell infiltration [5]. Protein unfolding induced by ischemia causes the activation of heat shock proteins (HSPs), which besides their well-known protective properties also show a pro-inflammatory effect [6,7]. DAMPs, acting mainly through RAGE and TLR receptors, activate microglia, astrocytes and endothelium, increasing the production of metalloproteinases and pro-inflammatory factors. This results in blood–brain barrier (BBB) breakdown and the infiltration of immune cells into the affected brain area. Further development of the inflammatory reaction is regulated by pro-inflammatory cytokines, such as IL1α and β, IL6, IL8 and IL17α and tumor necrosis factor alpha (TNFα) [8]. Additionally, S100 calcium-binding proteins, classified as DAMPs, also have chemotactic functions for immune cells [9].

The secretion of DAMPs into the surrounding environment strongly enhances the activation and polarization of resident microglia to an M1 or M2 phenotype. The proportion of M1 and M2 microglia tilts towards the M1 phenotype in the acute and subacute phases, beginning from day 3 [10]. Active microglia, which have a detrimental effect on the BBB at the onset of stroke, present a beneficial effect on stroke recovery in the subacute and chronic phases, facilitating tissue repair and wound healing. Moreover, some cytokines, which initially act in an inflammatory role, then contribute to the reconstruction of the brain’s blood vessels [8,11,12,13].

Studies on animal models are crucial for better understanding the molecular processes taking place during neurological diseases, being a solid basis for examining the effectiveness of various therapies. Due to the lack of access to human material to examine the DAMP expression in the brain tissue after ischemic stroke, research in humans is primarily focused on correlating the DAMP levels in blood serum with stroke occurrence and prognosis [2,14,15]. For this reason, rodent models have been extensively used to investigate the complex role of DAMPs, especially their involvement in the time-dependent influx of immune cells to the ischemic area and the subsequent inflammatory reaction [5,16]. However, studies on DAMPs in large animal models remain scarce. The porcine ischemic stroke model presents a unique and highly relevant platform for such research, as pigs possess a large gyrencephalic brain, a white/gray matter ratio comparable to that of humans and an immune system with greater translational relevance [17,18].

Studying the interactions of different DAMPs in the porcine brain following ischemic stroke will allow the identification of the key time points associated with the inflammatory response and initial signs of infarct site remodeling, which may provide a basis for the development of targeted therapies to support post-stroke recovery.

## 2. Results

### 2.1. Confirmation of Ischemic Stroke Induction

**Figure 1:**
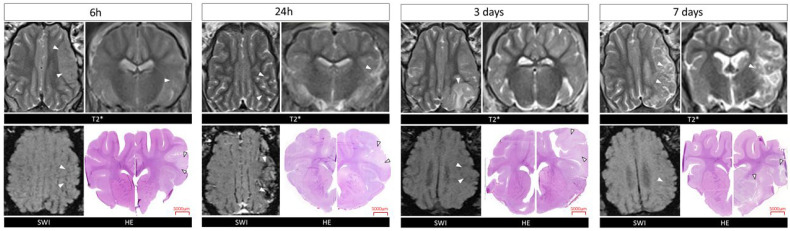
MRI and histological evaluation of stroke in Pig brains at 6 h, 24 h, 3 and 7 days. T2w SWI scans and HE images of brain tissue at different time points. White arrows indicate the site of the stroke. T*-T2 weighted scans.

MRI scans in T2 and SWI sequences confirmed the formation of ischemic stroke in each examined time interval. The location and area of the stroke shown by HE staining corresponded with those seen on MRI scans (Figure 1).

### 2.2. Purines and Calcium-Binding Proteins

**Figure 2:**
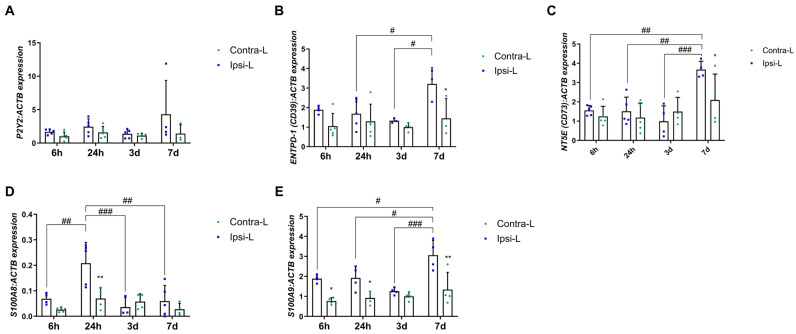
Gene expression of P2Y2 (**A**), ENTPD-1 (CD39) (**B**), NT5E (CD73) (**C**) and calcium-binding proteins—S100A8 (**D**) and S100A9 (**E**)—in the ipsilateral (ipsi-L, green spots) and contralateral (contra-L blue squares) hemisphere 6 h, 24 h, 3 days and 7 days after the onset of stroke. Statistical analysis: * *p* < 0.05, ** *p* < 0.01 (ipsilateral vs. contralateral hemisphere); # *p* < 0.05, ## *p* < 0.01, ### *p* < 0.001 (comparison between time points). Data are presented as mean ± SEM.

The gene expression of the G-protein-coupled receptor, *P2Y2*, was not affected by the ischemic stroke (Figure 2A; *p* > 0.05). The *ENTPD-1* (*CD39*) expression was elevated in the ipsilateral hemisphere 7 days post-stroke compared to in the contralateral hemisphere and compared to days 1 and 3 (Figure 2B; *p* < 0.05). Similarly, the *NT5E* (*CD73*) gene expression level was increased in the ipsilateral hemisphere at the end of the experiment compared to the previous days (*p* < 0.01 and *p* < 0.001) and compared to in the contralateral hemisphere (Figure 2C; *p* < 0.05). Calcium-binding proteins—S100A8 and S100A9—were significantly elevated in the stroke-covered hemispheres. The expression of S100A8 increased 24 h after stroke induction in the ipsilateral hemisphere vs. the contralateral hemisphere (*p* < 0.01) and versus all the other days of the experiment (Figure 2D; *p* < 0.01 for 6 h and 7 d and *p* < 0.001 for 3 d). Notably, S100A9 was elevated in the ipsilateral hemisphere on day 7 post-stroke compared to 6 h, 24 h and 3 d (*p* < 0.05, *p* < 0.05 and *p* < 0.001, respectively). Moreover, a significantly increased expression of S100A9 was noticed in the ipsilateral hemisphere vs. the contralateral hemisphere in 6 h, 24 h and 7 d experimental groups (Figure 2E, *p* < 0.05, *p* < 0.05 and *p* < 0.01, respectively).

### 2.3. Peroxiredoxins

**Figure 3:**
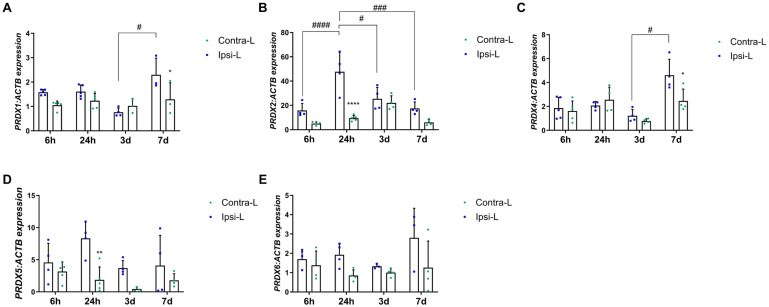
Gene expression of peroxiredoxins PRDX1 (**A**), PRDX2 (**B**), PRDX4 (**C**), PRDX5 (**D**) and PRDX6 (**E**) in the ipsilateral (ipsi-L, green spots) and contralateral (contra-L, blue squares) hemisphere 6 h, 24 h, 3 days and 7 days after the onset of stroke. Statistical analysis: * *p* < 0.05, ** *p* < 0.01, **** *p* < 0.0001 (ipsilateral vs. contralateral hemisphere); # *p* < 0.05, ### *p* < 0.001, #### *p* < 0.0001 (comparison between time points). Data are presented as mean ± SEM.

Peroxiredoxins are widely known for their antioxidant function. Nevertheless, they also control cytokine-dependent peroxide levels, which in stroke can lead to both the reduction in and the induction of inflammation. In our study, we observed an increased PRDX2 and PRDX5 gene expression in the hemisphere affected by stroke 24 h post-induction (Figure 3B,D; *p* < 0.0001 and *p* < 0.01, respectively). Moreover, the PRDX2 expression 24 h post-stroke was enhanced compared to the 6 h, 3 d and 7 d groups (*p* < 0.0001, *p* < 0.05 and *p* < 0.001, respectively). The gene expression of PRDX1 and PRDX4 was elevated on day 7 in the hemisphere affected by stroke in comparison with the contralateral hemisphere and compared to day 3 (Figure 3A,C; *p* < 0.05). There were no significant differences in the level of PRDX6 in all the studied time intervals (Figure 3E).

### 2.4. HMGB1, RAGE and TLR Receptors, TNFα and Transcription Factors

**Figure 4:**
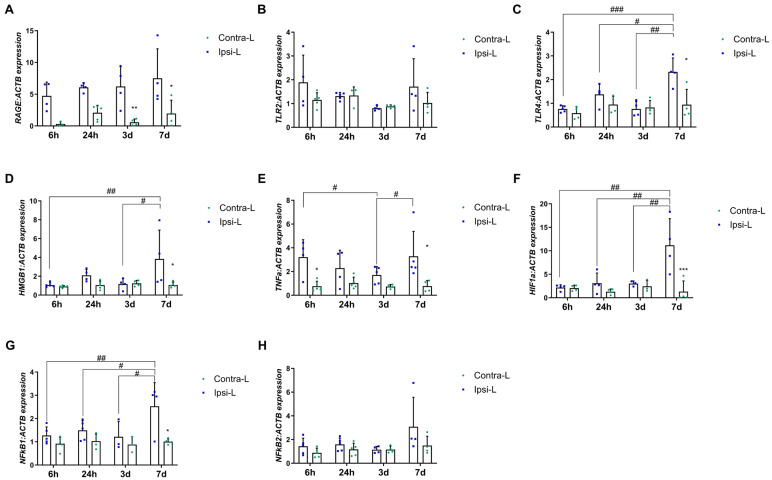
Gene expression of RAGE (**A**), TLR2 (**B**), TLR4 (**C**), HMGB1 (**D**), TNFα (**E**), HIF1α (**F**), NFκβ1 (**G**) and NFκβ2 (**H**) in the ipsilateral (ipsi-L, green spots) and contralateral (contra-L, blue squares) hemisphere 6 h, 24 h, 3 days and 7 days after the onset of stroke. Statistical analysis: * *p* < 0.05, ** *p* < 0.01, *** *p* < 0.001 (ipsilateral vs. contralateral hemisphere); # *p* < 0.05, ## *p* < 0.01, ### *p* < 0.001 (comparison between time points). Data are presented as mean ± SEM.

We observed the upregulation of the RAGE gene expression in the ipsilateral hemisphere vs. the contralateral hemisphere on days 3 and 7 post-stroke (Figure 4A, *p* < 0.01 and *p* < 0.05, respectively). The expression of HMGB1 was elevated in the ipsilateral hemisphere 7 days after stroke induction compared to in the contralateral hemisphere (*p* < 0.05) and compared to the 6 h and 3 d groups (Figure 4D; *p* < 0.01 and *p* < 0.05, respectively). Similarly, the expression of TLR4 and HIF1α was increased on day 7 in the ipsilateral hemisphere vs. the contralateral hemisphere (*p* < 0.05 and *p* < 0.001 for TLR4 and HIF1α, respectively) and versus other experimental groups (Figure 4C,F; *p* < 0.001, *p* < 0.05 and *p* < 0.001 for TLR4 and *p* < 0.01 for HIF1α).

Furthermore, the gene expression of TNFα was significantly elevated in the ipsilateral hemisphere at both 6 h and 7 days post-stroke induction when compared to in the contralateral hemisphere (Figure 4E; *p* < 0.05). No significant difference was observed in the TLR2 expression between the post-stroke hemisphere and the control at any of the time intervals examined (Figure 4B).

The NF-κβ21 gene expression was found to have increased 7 days post-stroke induction in the collapsed hemisphere compared to in the control hemisphere (*p* < 0.05) and compared to the 6 h, 24 h and 3 d groups (Figure 4G; *p* < 0.01, *p* < 0.05 and *p* < 0.05, respectively). The NF-κβ2 mRNA level was not changed in any of the analyzed time periods (Figure 4H).

### 2.5. Interleukins

#### 2.5.1. Gene Expression in the Brain Tissue

**Figure 5:**
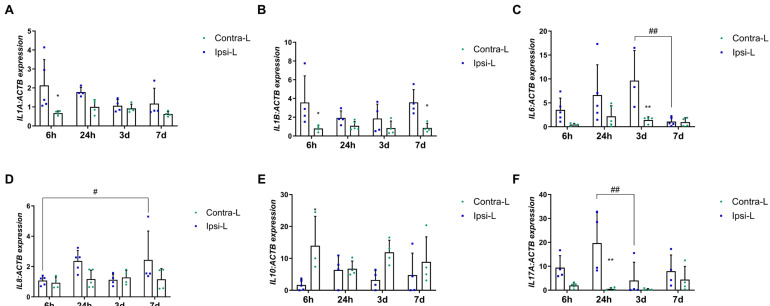
Gene expression of interleukins: IL1α (**A**), IL1β (**B**), IL6 (**C**), IL8 (**D**), IL10 (**E**) and IL17α (**F**) in the ipsilateral (ipsi-L, green spots) and contralateral (contra-L, blue squares hemisphere) 6 h, 24 h, 3 days and 7 days after the onset of stroke. Statistical analysis: * *p* < 0.05, ** *p* < 0.01 (ipsilateral vs. contralateral hemisphere); # *p* < 0.05, ## *p* < 0.01 (comparison between time points). Data are presented as mean ± SEM.

Cytokines as one of the first secreted DAMPs, but also as mediators of inflammation depending on their type, determine their development or inhibition after a stroke. The increase in the gene expression of pro-inflammatory interleukin IL1α was observed 6 h after stroke induction compared to in the contralateral hemisphere (Figure 5A,B; *p* < 0.05). Similarly, the IL1β expression was elevated at 6 h as well as 7 days post-stroke induction (Figure 5B; *p* < 0.05). The increase in the mRNA expression of one of the most important pro-inflammatory interleukins—IL6—was found 3 days after stroke induction compared to in the contralateral hemisphere (*p* < 0.01) and to day 7 (Figure 5C; *p* < 0.01). The significantly enhanced expression of IL8 was determined on day 7 compared to on 6 h in the ipsilateral hemisphere (Figure 5D, *p* < 0.05). The down-regulation of the IL10 mRNA expression was noticed in the ipsilateral hemisphere vs. the contralateral hemisphere 6 h after stroke induction (Figure 5E, *p* < 0.05). The gene expression of IL17α elevated significantly in the collapsed hemisphere 24 h after stroke induction compared to in the contralateral hemisphere (*p* < 0.01) and compared to day 3 (Figure 5F; *p* < 0.01).

#### 2.5.2. Concentration in the Blood Serum

**Figure 6:**
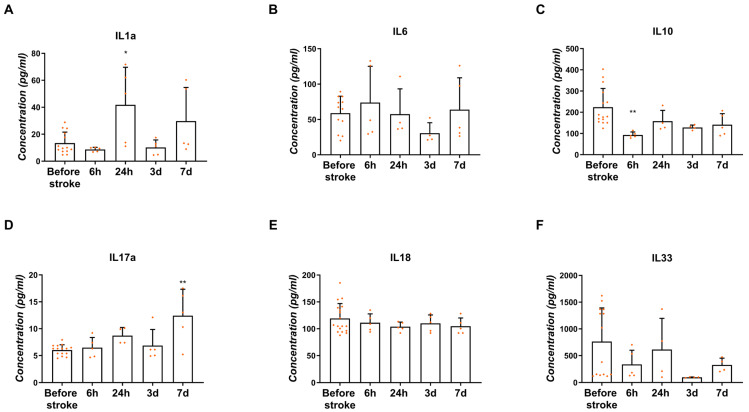
Concentration of interleukins IL1α (**A**), IL6 (**B**), IL10 (**C**), IL17α (**D**), IL18 (**E**) and IL33 (**F**) measured in the blood serum before the stroke and 6 h, 24 h, 3 days and 7 days after the onset of stroke. Statistical analysis: * *p* < 0.05, ** *p* < 0.01 (ipsilateral vs. contralateral hemisphere). Data are presented as mean ± SEM.

The concentration of interleukins in the blood serum collected before the stroke and in different time points after the stroke was measured with the use of the ELISA method. The concentration of IL1α increased significantly 24 h after stroke (39.71 ± 15.86 pg/mL) (Figure 6A; *p* < 0.05). Similarly, the IL1α concentration was around two times higher 7 days after stroke than before stroke; however, this was without statistical significance (34.04 ± 13.3 pg/mL) (Figure 6A). A relatively stable concentration of IL6 in the blood serum was noticed before as well as after the stroke (46.14–85.67 pg/mL) (Figure 6B). The mean concentration of IL10 decreased significantly 6 h after stroke (92.03 ± 7.83 pg/mL) in comparison to the blood serum before stroke (Figure 6C; *p* < 0.01). The increased concentration of IL17α was observed on the 7th day post-ischemia (11.46 ± 3.24 pg/mL) compared to the concentration before the stroke (6.03 ± 0.25 pg/mL) (Figure 6D; *p* < 0.01). No significant differences in the IL18 level were noticed before and after ischemic stroke (104.2–111.2 pg/mL) (Figure 6E). However, without statistical significance, a lower concentration of IL33 was noticed on day 3 (100.4 ± 1.6 pg/mL) compared to the mean concentration in the blood serum before the stroke (763.4 ± 168 pg/mL) (Figure 6F).

### 2.6. Heat Shock Protein Family

**Figure 7:**
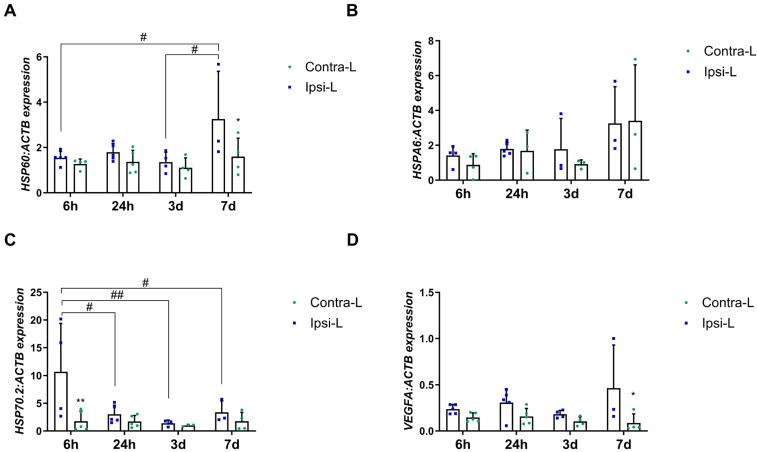
Gene expression of HSP60 (**A**), HSPA6 (**B**), HSP70.2 (**C**) and VEGF (**D**) in the ipsilateral (ipsi-L, green spots) and contralateral (contra-L, blue squares) hemisphere 6 h, 24 h, 3 days and 7 days after the onset of stroke. Statistical analysis: * *p* < 0.05, ** *p* < 0.01 (ipsilateral vs. contralateral hemisphere); # *p* < 0.05, ## *p* < 0.01 (comparison between time points). Data are presented as mean ± SEM.

Heat shock proteins are known for their neuroprotective role against cells after the onset of local ischemia or inflammation. In our study, we examined the expression of representants of the HSP60 and HSP70 families, as the most important in the ischemic brain. The increase in the HSPD1 (HSP60) gene expression was found in the stroke-affected hemisphere after 7 days when compared to in the contralateral hemisphere (*p* < 0.05) and compared to the 6 h and 3 d group (Figure 7A; *p* < 0.05). The isoform HSP70.2 gene expression was elevated 6 h post-stroke induction compared to in the contralateral hemisphere (*p* < 0.01) and all other time groups (Figure 7C; *p* < 0.05 for 24 h and 7 d groups, and *p* < 0.01 for 3 d). There were no significant changes in the HSPA6 (HSP70) expression between hemispheres in all the studied time intervals (Figure 7B).

### 2.7. Vascular Endothelial Growth Factor A (VEGFA)

Although VEGFA does not belong to the group of DAMPs, determining its expression is important in the context of the pro-angiogenic changes that occur after stroke. In our study, the enhanced expression of VEGFA was observed in the ipsilateral hemisphere 7 days post-stroke, compared to in the contralateral hemisphere (Figure 7D, *p* < 0.05).

## 3. Discussion

**Figure 8:**
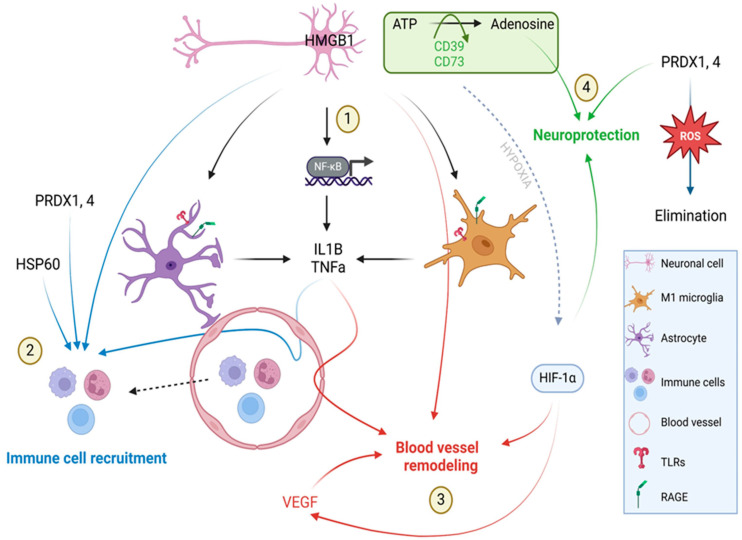
Schematic representation of DAMP action on 7 days after ischemic stroke. HMGB1 produced by necroptotic neuronal cells acting through RAGE and TLRs receptors and astrocytes and microglia activate the HMGB1–NF–κβ–pro-inflammatory cytokine axis leading to the enhanced production of IL1β and TNFα. Both cytokines (IL1β and TNFα) together with PRDX1,4 and HSP60 recruit immune cells to the infraction site. HMGB1, IL1β, TNFα and VEGFA facilitate post-stroke angiogenesis. Hypoxia induces the activation of HIF1α transcription factor which plays a role in vessel remodeling. Ectonucleotidases CD39 and CD73 hydrolyze extracellular ATP to adenosine, which has neuroprotective properties. PRDXs by the elimination of reactive oxygen species also play a neuroprotective role. Created with BioRender.com.

Cerebral ischemia resulting from an infarction of one of the arteries supplying the brain leads to the cascade of inflammatory responses initiated by the increased release of DAMPs, which, acting through receptors on microglia and astrocytes, cause the enhanced production of cytokines and the infiltration of immune cells from the periphery (Figure 8). One of these factors that plays an important role shortly after the initiation of ischemia is ATP [19], which was found to be released as early as 30 min after transient MCAO (tMCAO) in mice [20]. Studies on the rat tMCAO model revealed the upregulation of P2Y2 (one of the ATP receptors) expression one day after ischemia and suggested its role in the mitigation of brain damage by inhibiting YAP phosphorylation and reducing mitochondrial fission [21]. As we determined in the current study, the expression of P2Y2 is stable during the first seven days after ischemic stroke in pigs. However, there is a significantly higher expression of two ectonucleotidases (ENTPD-1, CD39 and NT5E, CD73) in the ipsilateral hemisphere 7 days after stroke. For over 30 years, research has been conducted to confirm the protective role of CD39 and CD73 in the brain affected by stroke. In a tMCAO mouse model, the administration of CD39 resulted in a significant reduction in the infarct size while improving blood circulation in the area [22]. Knockout of CD39 [22] as well as CD73 [23] in mice resulted in an increased infarct volume after stroke. Therefore, it is likely that the significant increase in the CD39 and CD73 gene expression one week after cerebral ischemia in pigs and in consequence an increase in extracellular adenosine may ameliorate the inflammatory response, playing a neuroprotective role [19].

The situation is slightly different in the case of the other members of DAMPs, i.e., proteins belonging to the Ca^2+-^binding S100 family. In our study, the enhanced expression of the S100A8 and S100A9 genes was already noticed in the ipsilateral hemisphere 24 h after stroke. It was previously suggested that the interaction of the S100A8 and S100A9 proteins with the TLR4 and RAGE receptors might be involved in brain damage and inflammation [24,25]. The size of the brain damage after ischemia was indicated to be related to the S100a8 level in mice [25], whereas the elevated level of the S100A8/A9 heterodimer was indicated in human plasma after ischemia and was suggested to serve as a prognostic of ischemic stroke [26]. Since S100A8 and S100A9 also exhibit chemotactic properties by recruiting immune cells, it can be assumed that their increased expression 24 h after stroke in pigs also contributes to the development of the inflammatory response [10].

Degenerating tissue, a lack of blood supply and developing inflammation also led to oxidative stress by the excessive production of reactive oxygen species (ROS). This leads to lipid peroxidation, protein oxidation and DNA fragmentation, causing enhanced brain damage [27,28]. The neuroprotective effect of peroxiredoxins (also categorized as DAMPs) is related to the elimination of ROS and lasts until they are released from the cell [5]. PRDX2 and PRDX6 have been shown to have a positive effect on brain tissue after stroke by reducing the level of ROS and thus its harmful effects and mitigating neuronal apoptosis in rats and dogs [29,30,31]. In our study, an enhanced expression of other PRDXs, PRDX1 and PRDX4, was determined one week after ischemia, allowing such a protective role to be presumed. On the other hand, the PRDXs produced by necrotic neurons stimulated macrophages for pro-inflammatory cytokine production and the resulting neuronal damage [29]. Studies in rats demonstrated the release of PRDXs into extracellular space within 12–24 h after ischemic stroke onset [31]. In current study, the enhanced expression of PRDX2 and PRDX5 was noticed in the ipsilateral hemisphere 24 h after stroke onset, which may suggest the pro-inflammatory role of those two PRDXs after ischemia, which is consistent with Shichita et al.’s study where PRDX blocking in mice resulted in a better outcome after stroke and was associated with the reduction in cells producing inflammatory mediators [32,33]. Interestingly, studies on humans showed that the plasma concentration of PRDX5 was negatively correlated with the biomarkers of inflammation and the severity of stroke [15]. Taken together, all these data point to the complexity of the functions performed by PRDXs after stroke and suggest the need for further, more detailed studies to determine the exact role of each.

After ischemic stress, the unfolding of proteins leads to the activation of stress-induced heat shock proteins leading to proteins refolding and preventing more extended tissue damage [34]. In the current study, we determined the enhanced expression of the HSP70.2 isoform shortly after stroke induction, which relates to its modulating role in mediating the immune response. It ensures the balance between the anti-inflammatory action of intracellular HSP70 and the intensifying immune response by extracellular HSP70 [35]. Moreover, in our study, the up-regulation of HSP60 was noticed 7 days after ischemia a few days later than during transient MCAO in rats, where enhanced expression occurred one day after reperfusion [36,37]. The enhanced expression of HSP60 on day 7 in pigs corresponded with HIF1α, NF-κβ and TNFα allowing for the presumption of a role for HSP60 in maintaining or triggering the inflammatory response after stroke.

We determined the elevated gene expression of pro-inflammatory cytokines such as IL1α and IL1β and TNFα as soon as 6 h after stroke induction. This points to the intensive and robust inflammatory response in the hyperacute phase of ischemic stroke, which is most likely related to the passive release of DAMPs by necroptotic neuronal cells. Another source of pro-inflammatory cytokines comprises the immune cells present in the infarction site. In most studies published on rodents, the infiltration of γδT cells and Th17 cells, which are the main sources of IL17α, was determined to peak three days after ischemia [37,38,39]. In our study, we noticed the up-regulation of IL17α gene expression one day after the stroke onset, whereas the protein content in blood serum was highest one week after the stroke. IL17α enlarges the inflammatory reaction through the chemotaxis of neutrophils to the injury site and promotes the death of neurons [38]. According to the available literature, neutrophils start to enter the parenchyma of a rodent’s brain 12 h after stroke onset, which peaks on days 2–7 [39]. However, the increased expression of the IL17α gene in ischemic pig brain tissue as early as 1 day after stroke may suggest earlier neutrophil infiltration in pigs. In the early subacute phase, i.e., 7 days after stroke, the concentration of IL17α in blood serum reached its peak, which correlated with the increased expression of HIF1α in the pig brain, supporting the theory that HIF1α is an activator of the transcription factor for IL17α (RORγt) [40]. Thus, it may be assumed that on day 7, the production of IL17α is enhanced due to the stimulation of the HIF1α-RORγt-IL17α axis in γδT cells and Th17 cells [41].

The increased expression of IL6 was observed three days after stroke onset in the hemisphere affected by the stroke. Increasing the tendency of IL6 expression from the very beginning of the acute phase is associated mainly with its release by the necroptotic neurons of the cerebral cortex [8,42]. Moreover, the microglial cells, astrocytes and endothelial cells are other sources of IL6 in the ischemic brain [8]. In humans, IL6 is a very important factor which serves as the marker of infarct size and post-stroke survival [43,44]. The role of IL6 varies between post-stroke phases; in the acute phase, IL6 has pro-inflammatory properties, whereas in the subacute and prolonged phase it acts as neurotrophic mediator [8].

The concentration of interleukins in the blood serum measured with the ELISA did not cover the profile of their gene expression in the brain tissue except IL10, which was decreased immediately after stroke. Those discrepancies most probably can be explained by the local and systemic action of the mentioned factors. Interleukins produced by necroptotic neuronal cells or other cells resident in the damaged area can act locally to recruit immune cells to the ischemic brain and may also have a systemic action on peripheral organs [45], leading to the observed differences in the trend of the obtained results.

Another factor from the DAMP group, which can be released passively by necrotic cells or actively by activated immune cells, is HMGB1 with its dual role after ischemic stroke. Necrotic neuronal cells produce higher amounts of HMGB1, acting through TLR2, TLR4 and RAGE to stimulate microglia to produce pro-inflammatory factors and metalloproteinases resulting in enhanced inflammation and BBB breakdown [5]. Thus, the high concentration of HMGB1 has been considered as the marker of severity and a poor prognostic after stroke [3,46]. In our study, an increased expression of HMGB1, TLR4 and NF-κβ1 was observed in the subacute phase, one week after the onset of stroke. The demonstrated up-regulation of the mentioned axis may be related to the still ongoing inflammation, as suggested in the context of HSP60 up-regulation, immune cell recruitment and the beginning of blood vessel remodeling in the affected area. The activation of the HMGB1-TLR4-NF-κβ axis was shown to result in the enhanced production of pro-inflammatory cytokines such as IL1β, IL6 and TNFα [47]. It is known that these factors, in addition to their strong pro-inflammatory properties, positively influence the reconstruction of blood vessels [48,49]. In our study, the up-regulation of TNFα was determined on the 7th day post-stroke, which may indicate a relationship between the inflammatory reaction and intense vascular remodeling one week after ischemia in pigs. Similarly, in rodents, TNFα induced endothelial cell proliferation which reached maximum mitotic activity on day 7 [50]. As was previously determined, HMGB1 promoted blood vessel remodeling not only through the stimulation of microglia and endothelial cells to produce pro-angiogenic factors but also by inducing the migration of endothelial progenitor cells [51,52]. Moreover, we observed the enhanced expression of the HIF1α gene 7 days post-stroke in the hemisphere affected by ischemia. As indicated, HIF1α showed pro-angiogenic properties in the ischemic brain by the homing and sprouting of bone marrow endothelial progenitor cells via the CXCL12/CXCR4, HMGB1, VEGFA/Flk1 and Nrp1/Dll4 axes on the activated astrocytes [52]. Therefore, our results presenting upregulated VEGF expression on day 7 post-stroke may confirm HIF1α’s pro-angiogenic role.

In summary, our study determined and systematized for the first time the importance and complexity of DAMPs’ actions after cerebral ischemia in pigs and emphasized their dual role. The complexity of their action is particularly visible at the beginning of the subacute phase, where, according to the results of our study, the inflammatory reaction, initiation of angiogenesis and partial neuroprotection may occur simultaneously. Understanding the secretion of DAMPs in relation to the chronology of stroke phases may enable the design of immunomodulatory therapies to promote anti-inflammatory and remodeling factors while inhibiting the expression and secretion of factors that worsen the patient’s condition.

## 4. Materials and Methods

### 4.1. Experimental Animals

All animal experiments were approved by the University of Warmia and Mazury’s local ethics committee (45/2019, 25 June 2019) and were performed according to ARRIVE guidelines. Experiments were performed according to the EU Directive 2010/63/EU as well as Poland Act 2015/01/15 “Act on the protection of animals used for scientific or educational purposes”.

### 4.2. Surgery and Sample Collection

We followed the procedures used in our previous studies [17]. The operations were conducted in an operating room designed for large animals. The animals (*n* = 20) were divided into four groups according to the time of sacrifice (6 h, 24 h, 3 days and 7 days). Animals were pre-anesthetized with atropine (0.05 mg/kg i.m., Polfa, Warsaw, Poland), xylazine (3 mg/kg i.m., Vet-Agro, Lublin, Poland) and ketamine (6 mg/kg i.m., Biowet, Puławy, Poland) and anesthetized by propofol (5 mg/kg/h i.v., B.Braun Melsungen AG, Melsungen, Germany) and sevoflurane (1–3%, Abbvie, Warssaw, Poland). The animals received butorphanol every 4 h (0.2 mg/kg i.m., Zoetis, Warsaw, Poland). A Sheath Introducer (5F, Terumo, Warsaw, Poland) was placed transdermally in the femoral artery, which was identified using ultrasonography. Using this port, an endovascular catheter (Vertebral 5F, 110 cm, Balton, Warsaw, Poland) was placed and using hydrophilic guidewire (Merit Laureate, Merit Medical, Poznan, Poland) and was navigated to the arch of the aorta and then to the common carotid artery. The catheter was placed in the ascending pharyngeal artery (APA) proximally to the rete mirabile. Continuous flushing with heparinized (5000 U/l) saline was maintained to avoid occlusion of the catheter. The animals with a catheter secured in the APA were transferred to a 3 T MRI scanner (Magnetom Trio, Siemens, Warsaw, Poland). The protocol of the MRI included dynamic GE-EPI (TE/TR  =  30/1750) for the assessment of the trans-catheter cerebral perfusion, GE-EPI to monitor blood clotting, SWI (TE/TR  =  20/28), diffusion, T2w (TE/TR  =  83/5660) and T1w with contrast (TE/TR  =  3.69/20). Under a real-time MRI, guidance thrombin solution (200 U/300 µL 0.9% NaCl; Biomed, Krakoww, Poland) mixed with gadolinium contrast (Bayer, Berlin, Germany) agent (1:20) was injected intra-arterially via a catheter with a pulsatile mode over 1 min. Following the confirmation (validation) of stroke induction under MRI, the pigs were awakened and transferred to an animal facility where they remained under veterinary care until the follow-up MRI. Immediately after the MRI, the animals were euthanized with an overdose of pentobarbital. The MRI follow-up was performed 1 h before scarifying (5 h, 23 h, 3 days, 7 days) post-stroke induction. All the vital parameters (saturation, heart rate, pressure, respiratory rate) were monitored during the entire procedure. The pig brain tissues were collected immediately after slaughter, and each hemisphere was divided into four parts, protected in liquid nitrogen (two of them) and stored at −80 °C for gene expression analysis or cryo-protected in 30% sucrose until sank, frozen on dry ice powder for five minutes and kept in −70 °C until further analysis. Hematoxylin and eosin staining for the evaluation of the stroke tissue was conducted according to Golubczyk et al. [17], as is revealed in Figure 1.

### 4.3. Hematoxylin and Eosin Staining

Tissue samples were cut on a Hyrax C25 PLMC cryostat (Zeiss, Oberkochen, Germany) at 10 μm thick coronal sections, placed on microscope slides and stored at −20 °C. The sections were dehydrated in an alcoholic series of EtOH (ethanol; 95%, 80%, 70% EtOH and distilled water) for 3 min each at room temperature. The sections were then washed in distilled water and stained for 10 min in hematoxylin solution and then for 10 min in running water. The sections were differentiated in acidified alcohol and washed for 5 min in running tap water. The sections were then transferred to an eosin solution for three minutes; washed in distilled water and rehydrated in 70%, 95% and 100% ethanol; then exposed in xylene (2 times for 10 min); and mounted using DPX medium (Merck KGaA, Darmstadt, Germany). Images were captured using a 3DHISTECH scanner (3DHISTECH Ltd., Budapest, Hungary).

### 4.4. Total RNA Extraction and Reverse Transcription

Total RNA extraction was conducted from brain tissues using a TRIzol^®^ reagent, according to the manufacturer instructions (Thermo Fisher Scientific, Inc., Waltham, MA, USA). The quality of RNA and its concentration were measured using a NanoDrop 1000 spectrophotometer (Thermo Fisher Scientific Inc., Waltham, MA, USA). An A260/A280 ratio of measured RNA had a value of 1.9 to 2.03. The integrity of RNA (RIN) was determined using Agilent Bioanalyzer 2100 (Agilent Technologies, Santa Clara, CA, USA). The RIN values ranged from 8.5 to 9.8. Subsequently, reverse transcription reactions were conducted using the Reverse Transcription System Kit (Applied Biosystems, Foster City, CA, USA). Two types of RT controls were used, one without RNA and another in the absence of the reverse transcriptase.

### 4.5. Quantitative Real-Time Polymerase Chain Reaction (qPCR)

The cDNA obtained was used for real-time quantitative PCR analysis using the Light Cycler 480 System (Roche, Basel, Switzerland). Each sample contained 3 μL (50 ng) cDNA, 1.5 μL RNAse-free water (Life Technologies, Carlsbad, CA, USA), 5 μL TaqMan Universal MasterMix II (Life Technologies, USA) and 0.5 μL TaqMan assays (Life Technologies, USA; listed in Table 1). The PCR conditions included the following: an initial denaturation step (10 min at 95 °C), followed by 50 cycles of denaturation (15 s at 95 °C) and annealing (60 s at 60 °C). Data obtained from the Real-Time PCR were normalized using the ratio of mRNA of examined genes to the β-actin mRNA. The housekeeping gene was chosen from four tested genes: GAPDH, β-actin, HPRT and SDHA, based on RefFinder [53]. The quantification of gene expression was performed using the comparative CT method.

### 4.6. ELISA

Blood samples were collected from animals before the stroke induction and after the slaughter. Serum samples were obtained by centrifugation at 3000× *g* for 10 min and stored in −80 °C. The ELISA was performed according to the manufacturer’s instructions. Each sample was run in duplicate. The following ELISA kits were used: IL1α (Biorbyt, Cambridge, UK, orb390929), IL6 (ELK Biotechnology, Wuhan, China, ELK5741), IL10 (EIAab, Wuhan, China, E0056p), IL17α (ELK Biotechnology, ELK5741), IL18 (Biorbyt, 390934), IL33 (Biorbyt, orb390944). The absorbance at specified wavelengths was measured on a microplate reader (Multiscan FC, Thermo Fisher Scientific).

### 4.7. Statistical Analysis

Statistical analyses were performed using GraphPad Prism 8.0 (GraphPad Software, Inc., San Diego, CA, USA). The distribution of normality was evaluated with a Shapiro–Wilk test. The two-way ANOVA followed by Turkey’s post hoc test was used to determine the differences in gene expression between the stroke-affected and control hemisphere and between different time intervals. One-way ANOVA followed by Turkey’s post hoc test was used to analyze the data from the ELISA. All the numerical data are presented as mean with standard deviation (SD), and differences were considered as statistically significant at the 95% confidence level (*p* < 0.05).

## Figures and Tables

**Table 1:** Accession numbers of probes used in Real-Time PCR analysis with gene ID.

Target Gene	Taq Man Probes	Gene ID
*IL1β*	Ss03393804	397122
*IL1α*	Ss03391335	397094
*IL6*	Ss03384604	399500
*IL8*	Ss03392437	396880
*IL10*	Ss03382372	397106
*IL17α*	Ss03391803	449530
*RAGE*	Ss03390846	396591
*HMGB1*	Ss03378573	445521
*TLR2*	Ss03381278	396623
*TLR4*	Ss03389780	399541
*TNF*	Ss03391318	397086
*HiF1α*	Ss03390447	396696
*PRDX1*	Ss06880613	100512476
*PRDX2*	Ss04327514	100512521
*PRDX4*	Ss06879099	100152260
*PRDX5*	Ss03394180	397273
*PRDX6*	Ss03384662	399538
*P2Y2*	Ss03378709	450248
*S100a8*	Ss04246257	100127488
*S100a9*	Ss04246618	100127489
*HSP60*	Ss01036749	492279
*HSPA6*	Ss03387784	396906
*HSP 70.2*	Ss03392270	396648
*NFKB1*	Ss03388575	751869
*NFKB2*	Ss06883748	100153829
*ENTPD1 (CD39)*	Ss03394207	397298
*NT5E (CD73)*	Ss06882629	100157995
*VEGFA*	Ss03393993	397157
*ACTB*	Ss03376563	414396
GAPDH	Ss03375629	396823
SDHA	Ss03376909	780433
HPRT1	Ss03388274	397351

## Data Availability

Data will be made available by the corresponding author upon request.
